# Ionizing Radiation Induces Altered Neuronal Differentiation by mGluR1 through PI3K-STAT3 Signaling in C17.2 Mouse Neural Stem-Like Cells

**DOI:** 10.1371/journal.pone.0147538

**Published:** 2016-02-01

**Authors:** Hyeon Soo Eom, Hae Ran Park, Sung Kee Jo, Young Sang Kim, Changjong Moon, Sung-Ho Kim, Uhee Jung

**Affiliations:** 1 Radiation Biotechnology Research Division, Advanced Radiation Technology Institute, Korea Atomic Energy Research Institute, Jeongeup, Republic of Korea; 2 Department of Biochemistry, College of Natural Sciences, Chungnam National University, Daejeon, Republic of Korea; 3 Department of Veterinary Anatomy, College of Veterinary Medicine, Chonnam National University, Gwangju, Republic of Korea; 4 Department of Radiation Biotechnology and Applied Radioisotope, University of Science and Technology (UST), Daejeon, Republic of Korea; Rutgers University, UNITED STATES

## Abstract

Most studies of IR effects on neural cells and tissues in the brain are still focused on loss of neural stem cells. On the other hand, the effects of IR on neuronal differentiation and its implication in IR-induced brain damage are not well defined. To investigate the effects of IR on C17.2 mouse neural stem-like cells and mouse primary neural stem cells, neurite outgrowth and expression of neuronal markers and neuronal function-related genes were examined. To understand this process, the signaling pathways including PI3K, STAT3, metabotrophic glutamate receptor 1 (mGluR1) and p53 were investigated. In C17.2 cells, irradiation significantly increased the neurite outgrowth, a morphological hallmark of neuronal differentiation, in a dose-dependent manner. Also, the expression levels of neuronal marker proteins, β-III tubulin were increased by IR. To investigate whether IR-induced differentiation is normal, the expression of neuronal function-related genes including synaptophysin, a synaptic vesicle forming proteins, synaptotagmin1, a calcium ion sensor, γ-aminobutyric acid (GABA) receptors, inhibitory neurotransmitter receptors and glutamate receptors, excitatory neurotransmitter receptors was examined and compared to that of neurotrophin-stimulated differentiation. IR increased the expression of synaptophysin, synaptotagmin1 and GABA receptors mRNA similarly to normal differentiation by stimulation of neurotrophin. Interestingly, the overall expression of glutamate receptors was significantly higher in irradiated group than normal differentiation group, suggesting that the IR-induced neuronal differentiation may cause altered neuronal function in C17.2 cells. Next, the molecular mechanism of the altered neuronal differentiation induced by IR was studied by investigating signaling pathways including p53, mGluR1, STAT3 and PI3K. Increases of neurite outgrowth, neuronal marker and neuronal function-related gene expressions by IR were abolished by inhibition of p53, mGluR-1, STAT3 or PI3K. The inhibition of PI3K blocked both p53 signaling and STAT3-mGluR1 signaling but inhibition of p53 did not affect STAT3-mGluR1 signaling in irradiated C17.2 cells. Finally, these results of the IR-induced altered differentiation in C17.2 cells were verified in *ex vivo* experiments using mouse primary neural stem cells. In conclusion, the results of this study demonstrated that IR is able to trigger the altered neuronal differentiation in undifferentiated neural stem-like cells through PI3K-STAT3-mGluR1 and PI3K-p53 signaling. It is suggested that the IR-induced altered neuronal differentiation may play a role in the brain dysfunction caused by IR.

## Introduction

Ionizing radiation (IR) is a good tool for cancer therapy on various tumors because it can easily penetrate into target areas located deep inside the organ without surgical operation [1]. In United States, brain tumors occupy 22% of tumors in young patients under 18 years of age and, approximately 30% of patients with solid tumors suffer from brain metastases [2]. Radiation therapy is very important remedy for brain tumors since chemotherapy and surgery are not applicable in many cases due to blood brain barrier and physical inaccessibility. However, normal tissues surrounding the cancer are also exposed to high doses of IR during radiotherapy. Thus, radiotherapy for brain tumors is sometimes accompanied by acute adverse effects, such as sickness, emesis, headache, vertigo and seizures, and late adverse effects such as cognitive deficits and memory loss [3]. Especially, the damage of a functionally important region in brain may cause severe complications limiting the outcome of radiotherapy.

Neurogenesis in mammalian brain is a serial process, including proliferation, migration, maturation and differentiation of neural stem cell (NSC) [4], and persists throughout life in only two areas, subgranular zone (SGZ) of dentate gyrus (DG) and subventricular zone (SVZ) of the lateral ventricles [5–7]. The impairment of cognition and learning and the loss of memory are well known as side effects of radiation therapy against brain tumors [8–10], and they are considerably attributed to damaged neurogenesis in SGZ and SVZ [11–14]. The actively dividing NSCs in these regions are very sensitive to IR [15]. Therefore, the decline of neurogenesis by IR could be resulted from the deficit of neural stem/precursor cells in SGZ and SVZ [16, 17]. In many studies, it has been reported that irradiation of rodent brain results in the decline of neurogenesis by loss of neuronal progenitor cells (NPC), being caused by cellular damage through oxidative stress, such as ROS, induced by radiation [18–23]. In addition, chronic inflammation is known to contribute to neurodegenerative change induced by IR [24–26]. It was reported that the chronic inflammation after irradiation was accompanied with increase of pro-inflammatory cytokines such as necrosis factor-alpha (TNF-α), interleukin-6 (IL-6) and interleukin-1 beta (IL-1β), and activation of microglia [27–31]. The consistent stimulation by activated microglia-released proinflammatory cytokines and reactive oxygen species (ROS) can induce the degeneration of dopaminergic cells [31]. Thus, most studies on IR have been focused on the direct or indirect cellular damage caused by IR in neuronal cells and tissues in brain [18–23, 27–35]. Although some researchers have announced that the proliferation and differentiation of NSC were not affected by X-ray [36] and the X-ray may accelerate astrocytic differentiation from NSC [37, 38], the effects of radiation on neuronal differentiation are still largely unknown.

The action of glutamate as an excitatory neurotransmitter is mediated by its receptors which consist of two families; the ionotropic glutamate receptors (iGluRs) and the metabotropic glutamate receptors (mGluRs). The iGluRs are the ligand-gated ion channels regulating cation influx for excitatory synapses and they are subdivided into N-methyl-D-aspartate (NMDA), α-amino-3-hydroxy-5-methylisoxazole-4-propionic acid (AMPA) and kainate (KA) receptors [39, 40]. In general, the iGuRs mediate synaptic plasticity leading to a long-term potentiation (LTP) [41, 42]. In embryonic brain and neurogenic zone of adult brain, the iGluRs are highly expressed and involved in regulation of neural development and neurogenesis [43–49]. The mGluRs, G-protein coupled receptors, are subdivided into group I (mGluR1, 5), group II (mGluR2, 3) and group III (mGluR4, 6, 7, 8). The group I mGluRs are mainly found on post-synaptic neurons while the group II and groupIII mGluRs are found on pre-synaptic neurons in the nervous system [50, 51]. The group I mGluRs are known to affect the fate of NPC and NSC and the formation of hippocampus. It was reported that the mGluR5 plays a major role in survival and proliferation of NPC in vitro and in vivo [52, 53], and in regulation of human hippocampal development [54, 55], and the mGluR1 is involved in proliferation of NPC from adult mouse SVZ [56]. Also, recently it was found that mGluR1 and mGluR5 can increase differentiation of NSC [57].

Previously, we reported that Neuro-2a mouse neuroblastoma cells irradiated by γ-rays undergo morphological and molecular changes similar to neural differentiation [58]. Therefore, we hypothesized that the alteration of neuronal differentiation might be an important causing factor for IR-induced cognitive impairment. To address this question, we examined in this study whether IR can induce neuronal differentiation in C17.2 cells and primary NSCs, characterized the neuronal properties of differentiated cells, and identified the signaling pathways involved in this process. The morphological and molecular properties including neurite outgrowth and neuronal marker expression were examined. To identify whether neuronal differentiation by stimulation of IR or neurotrophin is different, the expressions of neuronal function-related genes including synaptophysin, synaptotagmin1, GABA receptors and glutamate receptors were examined. To confirm the molecular mechanism of the IR-induced altered neuronal differentiation, various key molecules known to be involved in neuronal differentiation were examined, including p53, PI3K, STAT3 and MAPKs.

## Materials and Methods

### Mice and isolation of mouse primary neural stem cells

Female C57BL/6 (H-2b) mice with 15 days of pregnancy were purchased from Damul Science (South Korea). The newborn C57BL/6 mice were sacrificed at one or two days after birth, and primary neural stem cells were isolated by enzymatic digestion of hippocampal regions of the brain. After euthanization of newborn mice with 300 mg/kg + 45 mg/kg ketamine/xylazine mix, the whole brain was immersed in Hank’s Balanced Salt Solution (HBSS; Sigma-Aldrich). Then, the hippocampal region was dissected from the brain under dissecting microscope (Leica, Wetzlar, Germany. The hippocampi pooled from about 10 pups were minced, washed twice in HBSS, and then dissociated in a digestion buffer containing 10 U/ml papain (Sigma-Aldrich), 0.45 mg/ml cysteine (Sigma-Aldrich) and 1000 U/ml DNase I (Sigma-Aldrich) in HBSS, pH 7.6, at 37°C for 30 min with a gentle shaking every 5 min. The digested tissues were washed in HBSS three times and then triturated in DMEM/F12 (Gibco) using a pipette. The resuspended single cells were used for the experiment. The animal experiment was performed according to the guidelines for the use and care of laboratory animals of Ministry of Health and Welfare (Korea), and was approved by the Institutional Animal Care and Use Committee of the Korea Atomic Energy Research Institute (KAERI).

### Cell culture

C17.2 mouse neural stem-like cells were obtained from American Type Culture Collection (ATCC, VI, USA) and cultured in Dulbecco’s modified Eagle’s medium (DMEM; Hyclone, UT, USA) supplemented with 10% (volume/volume) fetal bovine serum (FBS; Gibco, MD, USA), 5% horse serum (Gibco), 100 U/ml penicillin (Sigma-Aldrich-Aldrich, MO, USA), and 100 μg/ml streptomycin (Sigma-Aldrich) at 37°C in 5% CO_2_-humidified atmosphere. DMEM/F12 (Gibco) media with 1× N2 supplement (Gibco), 20 ng/ml nerve growth factor (NGF) (Gibco) and brain-derived neurotrophic factor (BDNF) (Gibco) were used in condition for differentiation.

The primary neural stem cells isolated from newborn mice were cultured in a growth media (1× N2 supplement (Gibco), 20 ng/ml epidermal growth factor (EGF; Gibco), 20 ng/ml basic fibroblast growth factors (bFGF; Gibco) and 2 μg/ml gentamycin (Sigma-Aldrich) in DMEM/F12 in a multi-well chamber slide (Corning, NY, USA) coated with poly-D-lysine (5mg/ml) (Sigma-Aldrich) at 37°C in a 5% CO_2_-humidified atmosphere [59]. The growth media were changed every other day.

### Irradiation and treatment of cells

1~3×10^5^ cells were plated in a 100 mm dish or multi-well chamber slide and incubated for 24 hr before irradiation. The cells in a culture dish were exposed to ^137^Cs γ-rays (0.95 Gy/min) at 1~16 Gy with Gamma Cell 40 Exactor (Nordion International Inc., Ontario, Canada) and the culture media were replaced with fresh media within 30 minutes. For p53 inhibition, PFT-α (Sigma-Aldrich) was added to the culture media at the final concentration of 20 or 40 μM 2 hr prior to the irradiation or p53-siRNA (Trp53 Silencer Select Pre-designed siRNA, ID: s75472; Invitrogen, CA, USA) was treated with Neuro-2a cells using Lipofectamine RNAiMAX (Invitorgen) using the manufacturer’s protocol at 24 hr prior to the irradiation. After incubation for 72 hr, the effect of PFT-α and p53-siRNA was identified through western blotting. For the experiments using inhibitors or antagonists on mitogen-activated protein kinase kinase (MEK) (PD98059; Cell Signaling Technologies, MA, USA), protein kinase A (PKA) (H-89; Cell Signaling Technologies), p38 (SB203580; Cell Signaling Technologies), PI3k (LY294002; Cell Signaling Technologies), tropomyosin receptor kinase A (Trk A) (AG879; Tocris, Bristol, UK), tropomyosin receptor kinase B (Trk B) (ANA12; Tocris), ataxia telangiectasia mutated (ATM) (KU55933; Selleckchem, TX, USA), ataxia telangiectasia and Rad3-related protein (ATR) (VE821; Selleckchem), c-Jun N-terminal kinase (JNK) (SP600125; Selleckchem), signal transducers and activators of transcription factors 1 (STAT1) (Fludarabine; Selleckchem), signal transducers and activators of transcription factors 3 (STAT3) (S3I-201; Selleckchem), GTPase (Rac) (NSC23766; Selleckchem), rapidly accelerated fibrosarcoma kinase (B-Raf) (GDC-0879; Selleckchem), metabotropic glutamate receptor 1 (mGluR1) (LY367385; Tocris), mGluR5 (MPEP; Tocris), Group II mGluRs (mGluR2 and mGluR3) (LY341495; Tocris) and Group III mGluRs (mGluR4, mGluR7 and mGluR8) (MSOP; Tocris), Neuro-2a cells were treated with these inhibitors at 5 or 10 μM and C17.2 cells were treated with theses inhibitors or antagonists at 0.1~25 μM, 2 hr prior to the irradiation. The inhibitors or antagonists used in this study are presented in Table 1.

**Table 1:** Inhibitors or antagonists used in this study.

Proteins	Inhibitors or Antagonists
p53	Pft-α
MEK	PD98059
PKA	H-89
p38	SB203580
PI3K	LY2940020
ATM	KU55933
ATR	VE821
Trk A	AG879
Trk B	ANA12
JNK	SP600125
STAT1	Fludarabine
STAT3	S3I-201
Rac	NSC23766
B-Raf	GDC-0879
mGluR1	Ly367385
mGluR5	MPEP
mGluR2/mGluR3	LY341495
mGluR4/mGluR7/mGluR8	MSOP

### Neurite outgrowth assay

Microscopic images of cells were captured using a DFC500 R2 digital camera (Leica, Wetzlar, Germany) at 72 hr after irradiation of Neuro-2a cells. To determine the rates of neurite-bearing cells, approximately 200 cells from three randomly taken images were analyzed each using Image J (ver. 1.46r) software (National Institutes of Health, MD, USA). The cells with neurite longer than the cell diameter were regarded as neurite-bearing cells [60].

### Western blot

The cells were lysed in a lysis buffer (50 mM Tris-Cl, pH 7.6, 150 mM NaCl, 1% NP-40, 25 mM NaF, 1 mM Na_3_VO_4_, 2 mM ethylene glycol tetraacetic acid (EGTA; Sigma-Aldrich), protease inhibitor cocktail (Sigma-Aldrich) for 30 min on ice. After centrifugation at 16000×g for 10 min, and 4°C for 15 min, the supernatant was collected, and its protein concentration was then measured using a bicinchoninic acid (BCA) protein assay kit (Thermo, IL, USA). Proteins (25–30 μg) were resolved by sodium dodecyl sulfate-polyacrylamide gel electrophoresis (SDS-PAGE) and transferred to a polyvinylidene fluoride (PVDF) membrane (GE Healthcare, Bucks, UK). Membranes were blocked in 5% skim milk (Sigma-Aldrich) or bovine serum albumin (BSA; Sigma-Aldrich), and incubated overnight at 4°C with antibodies of neuronal class III beta-tubulin (1:1000 dilution; Cell Signaling Technologies), nestin (1:1000 dilution; Cell Signaling Technologies), p53 (1:1000 dilution; Cell Signaling Technologies), phospho-p53 (p-p53) (1:1000 dilution; Cell Signaling Technologies), protein kinase B (AKT) (1:1000 dilution; Cell Signaling Technologies), phospho-AKT (p-AKT) (1:1000 dilution; Cell signaling Technologies), glial fibrillary acidic protein (GFAP) (1:1500 dilution; Cell signaling Technologies), mGluR1 (1:500 dilution; abcam, MA, USA), STAT3 (1:1000 dilution; Cell signaling Technologies), p-STAT3 (1:500 dilution; Cell signaling Technologies) and β-actin (1:2000 dilution; Cell Signaling Technologies). After washing with Tris-buffered saline-Tween 20 (TBST), the membranes were incubated with horseradish peroxidase (HRP)-conjugated anti-mouse immunoglobulin G (IgG) or anti-rabbit IgG antibody (1:2500 dilution; Cell Signaling Technologies) for 1 hr. The membranes were washed in TBST, and the immunoreactives were then detected using an enhanced chemiluminescence detection kit (Millipore, Darmstadt, Germany). The results were visualized and analyzed using a digital gel imaging system (EDAS290; Eastman Kodak Co., NY, USA).

### Total RNA isolation and quantitative real-time PCR

The total RNA was isolated using an easy-BLUE total RNA extraction kit (iNtRON Biotechnology, Seongnam-Si, Korea). A quantification and purity check of the RNA was performed based on an absorbance rate of A260/A280. 5 μg aliquots of total RNA were reverse transcribed into cDNA using moloney-murine leukemia virus (M-MLV) reverse transcriptase (Promega, WI, USA). The cDNA (1 μl RT product) was analyzed through real-time PCR using a StepOne Real-Time PCR system (Applied Biosystems, NY, USA) with SYBER Green TOPreal^™^ qPCR 2X PreMIX (Enzynomics, Daejeon, Korea). The gene primer sequences for real-time PCR are presented in Table 2. The amplification conditions of these genes were as follows: 95°C for 15 min, followed by 40 cycles at 95°C for 15 s, 60°C for 10 s, and 72°C for 15 s. The β-actin was used as a control for normalization.

**Table 2:** Primer sequences for real-time PCR used in this study.

Gene	Primer sequence	
GAP43	Forward	5’-AACGGAGACTGCAGAAAGCA-3’
	Reverse	5’-CTCATCCTGTCGGGCACTTT-3’
Rab13	Forward	5’-CTTCAACAGCACTTACATCT-3’
	Reverse	5’-CGGTAATAGGCGGTAGTTAT-3’
Synaptophysic	Forward	5’-GACGTTGGTAGTGCCTGTGA-3’
	Reverse	5’-GCACAGGAAAGTAGGGGGTC-3’
Synaptotagmin1	Forward	5’-GGGATGCTCTGACCGAGTTC-3’
	Reverse	5’-CCTCAAGTTGCCCACTGCTA-3’
GABA_A_R	Forward	5’-CTCTCCACACGTGTAAGCCT-3’
	Reverse	5’-AGCACAACCGATCCTTGTAA-3’
GABA_B_R	Forward	5’-ATTTCAGCAGCAGGGGTCTT-3’
	Reverse	5’-GGAAGAAGCAACATTTAAGG-3’
NMDA2a	Forward	5’-AGAACTCCACGCATTGCAGA-3’
	Reverse	5’-CATAGAGGTTCCCCATCCGC-3’
AMPA1	Forward	5’-GAAACTCAGTGAGCAAGGCG-3’
	Reverse	5’-CCAGCCCTCCAATCAGGATG-3’
mGluR1	Forward	5’-GAAACTAGAGACGGAGCGGG-3’
	Reverse	5’-GTCCGAACCAATCCCCATGT -3’
mGluR5	Forward	5’-TGGTGTTTGTGAAGCCAGTT-3’
	Reverse	5’-CAGGAAGCACCACTAGGACA-3’
mGluR2	Forward	5’-TTCTTTGCCTTTGCCGCTTC-3’
	Reverse	5’-CAGCAGTGCCAGGAACCTAA-3’
mGluR3	Forward	5’-GCTCCCTCTTTTGTGTCGGA-3’
	Reverse	5’-CTGGCAGGAACTGAAGCTGA-3’
mGluR4	Forward	5’-CGCCATTGCTGGCACATAAT-3’
	Reverse	5’-TCTTTTCCCCTGCTTTCCCC-3’
mGluR7	Forward	5’-GTAGACCCAAACAGCCCTGC-3’
	Reverse	5’-AGGTCTTCCTCCTCTGTGGTT-3’
mGluR8	Forward	5’-ATTGCCTGGCCTGAGTGAC-3’
	Reverse	5’-AGCAGAATTCTTCCTGGGCT-3’
β-actin	Forward	5’-GCAAGCAGGAGTACGATGAG-3’
	Reverse	5’-AGGGTGTAAAACGCAGCTCA-3’

### Immunocytochemistry (ICC)

The cells were fixed with 4% paraformaldehyde in phosphate-buffered saline (PBS) for 15 min and permeabilized in 0.5% Triton X-100 in PBS for 10 min at room temperature (RT). The cells were washed in PBS for 5 min and incubated with a blocking buffer containing 10% normal goat serum (Thermo sci., NY, USA), 5% BSA and 0.5% Tween 20 in PBS at RT for 2 hr. The cells were then incubated for 2hr at RT with primary antibodies of nestin (1:100 dilution, conjugated Cy3; Millipore) and β-III tubulin (1:400 dilution, conjugated Alexa; Millipore). The cells were washed for 10 min in PBS, and Hoechst 33258 (50 nM) (Sigma-Aldrich) was used for the counterstaining of nuclei. Fluorescent images were captured using a DFC500 R2 digital camera (Leica). To determine the ratio of β-III tubulin positive or nestin positive cells, approximately 300~500 cells from three to five randomly taken images were each analyzed by Image J (ver. 1.46r) software (National Institutes of Health).

### Statistical analysis

All data were presented as the mean ± standard deviation (SD) of triplicated experiments. The statistical significance was analyzed using an analysis of variance (ANOVA) followed by a Tukey’s test for multiple comparisons and 2-way ANOVA for the synergistic effects using SPSS statistic 22 (IBM, NY, USA). *p* < 0.05 was considered significant.

## Results

### Increase of neurite outgrowth and the related gene expressions by IR

**Fig 1:**
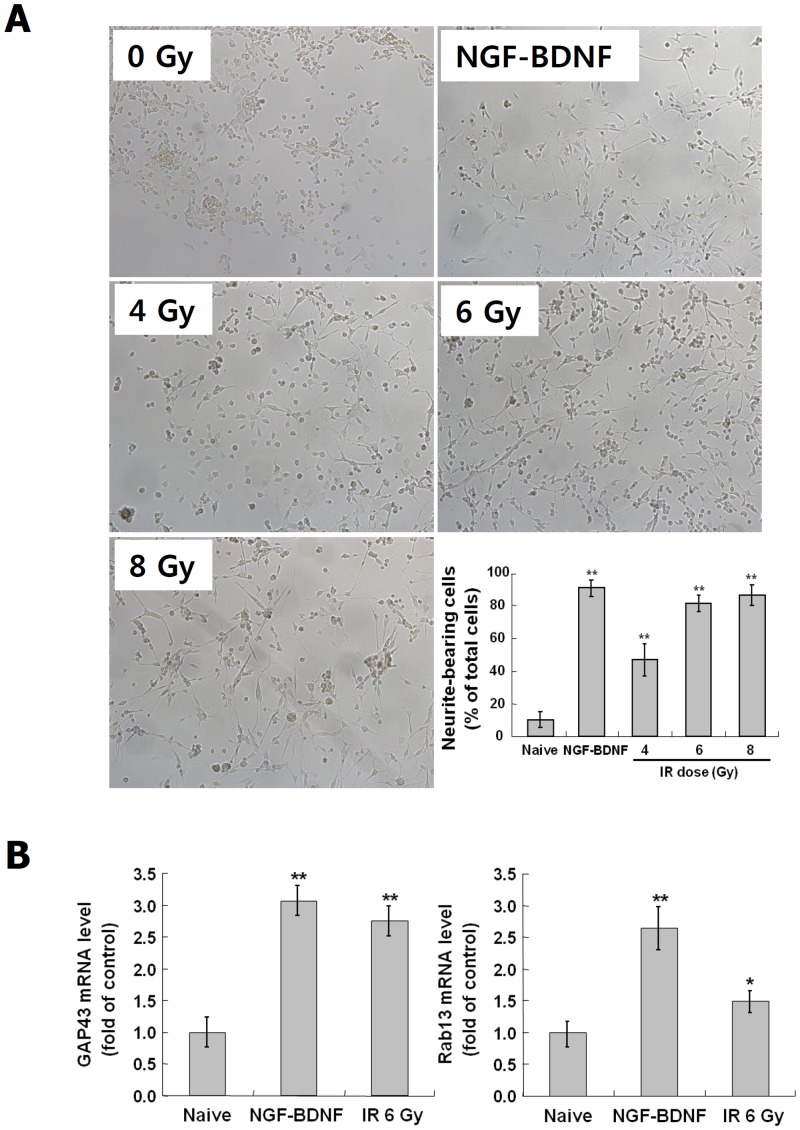
Induction of neurite outgrowth and the related genes by IR in C17.2 cells. C17.2 cells were exposed to IR at 4, 6 and 8 Gy or treated with NGF and BDNF at 20 ng/ml, and incubated at 37°C for 72 hr. The morphological change for neurite outgrowth by IR was observed in microscopic images (×200 magnification) (A). To assess rate of neurite-bearing cells, each 200 cells in three randomly taken images were analyzed by Image J software (A, right-down panel). To confirm IR-induced mRNA expression of neurite outgrowth-related genes, GAP43 and Rab13, irradiated Neuro-2a cells were analyzed by real-time PCR (B). The results represent the mean ± SD from triplicate data. *p < 0.05, **p < 0.01 vs naive group.

To evaluate IR-induced morphological properties in C17.2 mouse neural stem-like cells, neurite outgrowth and the expression of neurite outgrowth-related genes were examined. For comparison with normal differentiation, C17.2 cells treated with NGF and BDNF were also examined. The cells were exposed to IR at 4~8 Gy, or NGF/BDNF at 20 ng/ml, incubated for 72 hr and analyzed for neurite-bearing cells and the expression of GAP43 and Rab13 were examined. As shown in Fig 1A, the rate of neurite-bearing cells was significantly increased in NGF/BDNF-treated group (91%, *p* < 0.01) compared to naive control group (8%). The percentage of neurite-bearing cells was also raised by IR at 4 Gy (46%, *p* < 0.01), 6 Gy (81%, *p* < 0.01) and 8 Gy (85%, *p* < 0.01) in a dose-dependent manner. The expressions of GAP43 and Rab13 were markedly increased in NGF/BDNF-treated group (GAP43; 3 fold, *p* < 0.01 and Rab13; 2.6 fold, *p* < 0.01) and also in irradiated group at 6Gy (GAP43; 2.7 fold, *p* < 0.01 and Rab13; 1.5 fold, *p* < 0.05) (Fig 1B). These results showed that IR can induce a neuron-like morphological change of C17.2 cells similarly to neurotrophin treatment.

### Increase of neuronal marker expressions by IR

**Fig 2:**
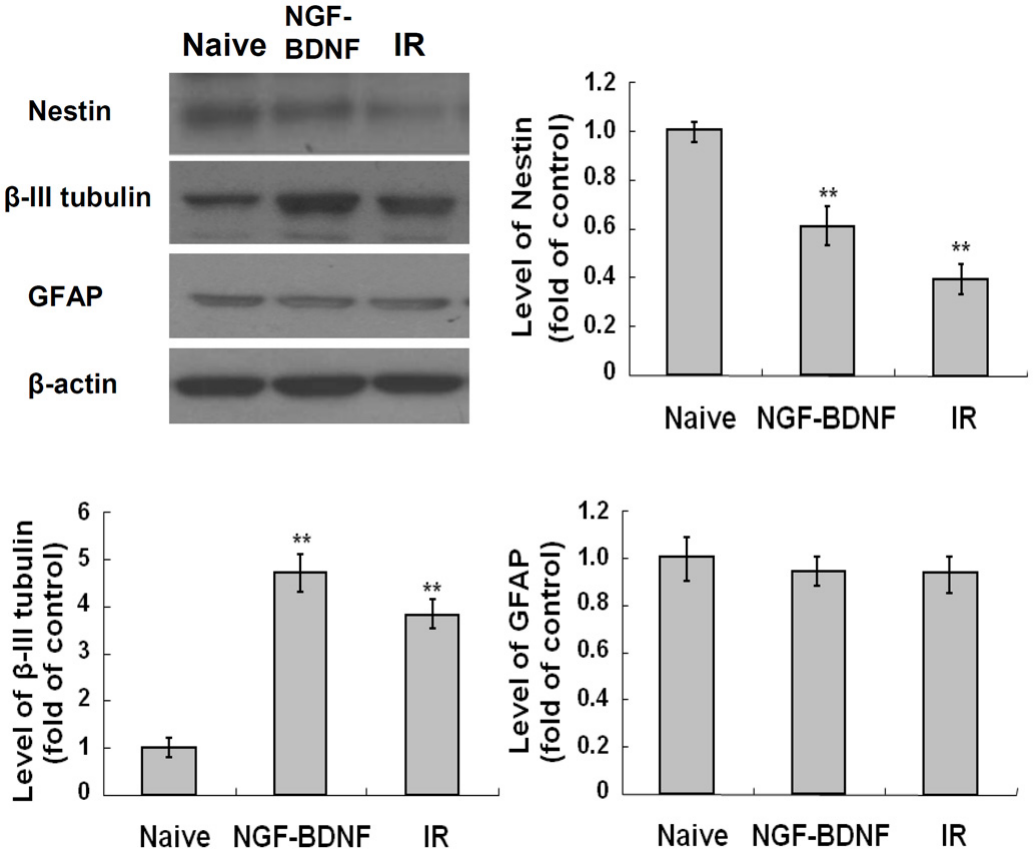
Induction of neuronal marker by IR in C17.2 cells. C17.2 cells were exposed to IR at 6 Gy or treated with NGF and BDNF at 20 ng/ml, and incubated at 37°C for 72 hr. Effects of IR on expression of NSC marker, nestin, neuronal marker, β-III tubulin, and astrocyte marker, GFAP were analyzed by Western blot and quantified by Image J software. The results represent the mean ± SD from triplicate data. **p < 0.01 vs naive group.

The IR-induced differentiation of C17.2 cells was identified by expression of NSC marker (nestin), neuronal marker (β-III tubulin) and astrocyte marker (GFAP). As shown in Fig 2, the expression level of nestin was markedly decreased in NGF/BDNF-treated group (0.6 fold, *p* < 0.01) and irradiated group at 6 Gy (0.4 fold, *p* < 0.01) compared naive control group. On the contrary, the expression level of β-III tubulin was significantly increased in NGF/BDNF-treated group (4.7 fold, *p* < 0.01) and irradiated group at 6 Gy (3.8 fold, *p* < 0.01). The expression of GFAP was not changed in both groups. These results suggest that C17.2 cells can differentiate into neuron-like cells when stimulated by IR similarly to neurotrophin treatment.

### Expression of synaptophysin, synaptotagmin1 and GABA receptors in irradiated C17.2 cells

**Fig 3:**
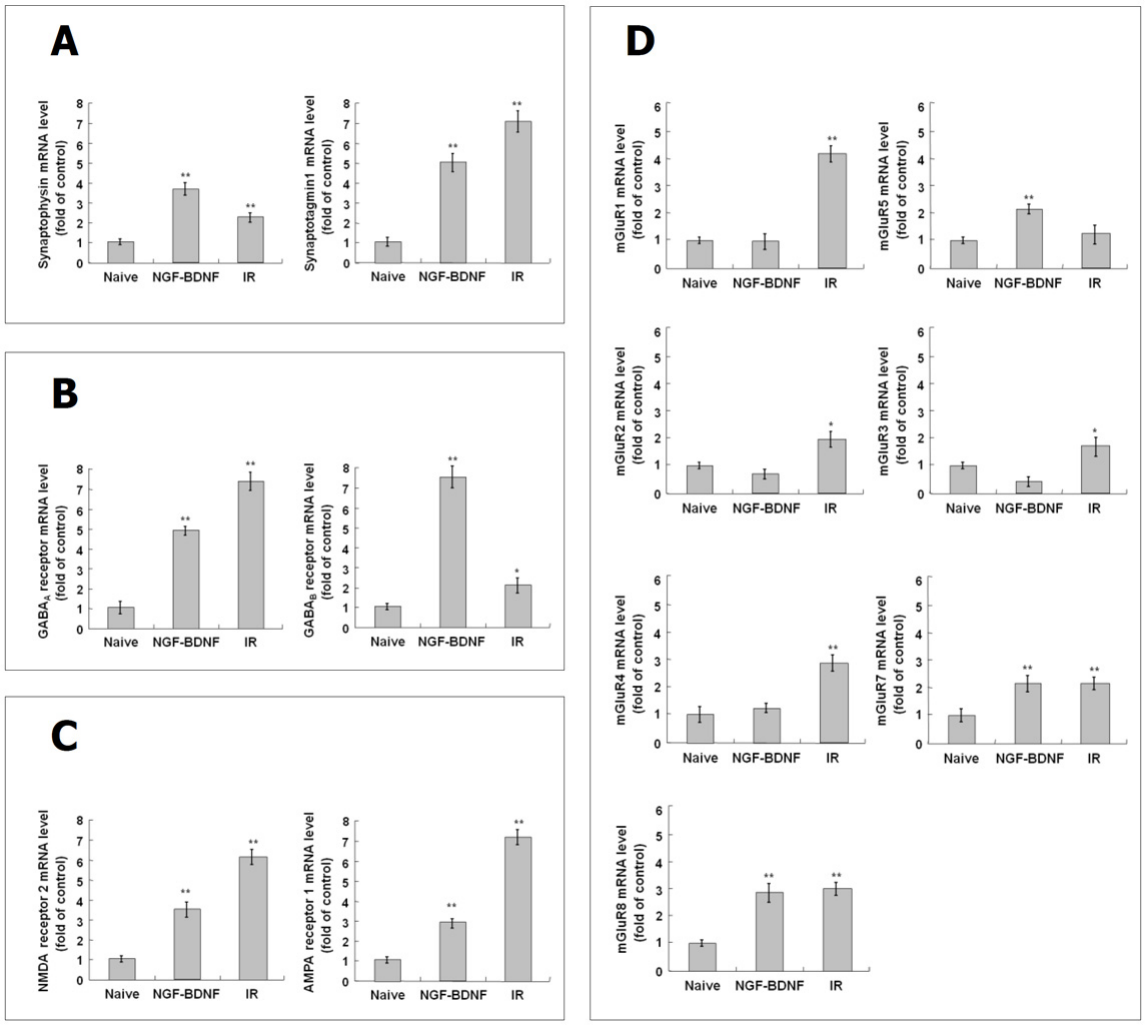
Induction of neuronal function-related genes by IR in C17.2 cells. C17.2 cells were exposed to IR at 6 Gy or treated with NGF and BDNF at 20 ng/ml, and incubated at 37°C for 72 hr. To assess effect of IR on neuronal function-related genes, expression of synaptophysin and synaptotagmin1 (A), GABA_A_ and GABA_B_ receptors (B), ionotropic glutamate receptors (C), and metabotropic glutamate receptors (D) was analyzed by real-time PCR. The results represent the mean ± SD from triplicate data. *p < 0.05, **p < 0.01 vs naive group.

To investigate whether the expression of neuronal function-related genes is induced by IR, the mRNA expressions of synaptophysin (a synaptic vesicle forming protein), synaptotagmin1 (a calcium ion sensor), and GABA_A_R and GABA_B_R (inhibitory neurotransmitter receptors) were examined in C17.2 cells in comparison with normal differentiation. The expression of synaptophysin was significantly increased in both NGF/BDNF-treated group (3.7 fold, *p* < 0.01) and irradiated group (2.2 fold, *p* < 0.01) at 6 Gy (Fig 3A). The expression of synaptotagmin1 was also markedly increased in both NGF/BDNF-treated group (5.1 fold, *p* < 0.01) and irradiated group at 6 Gy (7.1 fold, *p* < 0.01) (Fig 3A). The expression of GABA_A_R and GABA_B_R was considerably increased in NGF/BDNF-treated group (4.9 fold, *p* < 0.01 and 7.5 fold, *p* < 0.01) and in irradiated group at 6 Gy (7.4 fold, *p* < 0.01 and 2.2 fold, *p* < 0.05) (Fig 3B). These results suggest that IR can induce neuronal function-related gene expression in C17.2 cells similarl to neurotrophin treatment.

### Expression of excitatory neurotransmitter receptors in irradiated C17.2 cells

To investigate the effects of IR on the mRNA expression of ionotropic glutamate receptors (NMDA and AMPA) and metabotropic glutamate receptors (mGluR1~8) was examined in irradiated C17.2 cells in comparison with NGF/BDNF-treated cells. The expression of NMDA and AMPA was significantly raised in NGF/BDNF-treated group (3.5 fold, *p* < 0.01 and 2.9 fold, *p* < 0.01) and in irradiated group at 6 Gy (6.1 fold, *p* < 0.01 and 7.2 fold, *p* < 0.01) (Fig 3C). As shown in Fig 3D, the expression of mGluR5 was significantly increased in NGF/BDNF-treated group (2.1 fold, *p* < 0.01) but was not changed in irradiated group at 6 Gy. On the other hand, the expression of mGluR1, 2, 3 and 4 was not changed in NGF/BDNF-treated group but markedly raised in irradiated group at 6 Gy (4.2 fold, *p* < 0.01, 1.9 fold, *p* < 0.05, 1.7 fold, *p* < 0.01 and 2.9 fold, *p* < 0.01). The expression of mGluR7 and mGluR8 was increased in irradiated group (2.1 fold, *p* < 0.01 and 3.0 fold, *p* < 0.01) similarly to NGF/BDNF-treated group (2.1 fold, *p* < 0.01 and 2.9 fold, *p* < 0.01).

### An altered differentiation induced by IR in comparison to normal differentiation

The expression profiles of neuronal function related-genes in C17.2 cells were summarized and compared between irradiated cells and NGF/BDNF-treated cells. Among 13 genes analyzed in this study, 8 genes were expressed at higher level, 3 genes were expressed at lower level, and 2 genes were expressed at the similar level in IR-stimulated C17.2 cells compared to neurotrophin-stimulated cells, as shown in Table 3. Especially, among 9 genes of excitatory neurotransmitter receptors, 6 genes showed significantly higher expression level in IR-stimulated cells than neurotrophin-stimulated cells. From these results, it is suggested that IR can induce an altered neuronal differentiation of C17.2 cells compared to neurotrophin treatment.

**Table 3:** Summary of expression profile of neuronal function-related genes in IR-stimulated and neurotrophin-stimulated C17.2 cells.

		Relative expression compared to unstimulated control (fold)		
Functional Category	Gene name	NGF-BDNF stimulation	IR stimulation	Ratio ^a^
Synapse-related proteins	Synaptophysin	3.7 ± 0.2	2.2 ± 0.1*	0.6 (lower)
	Synaptotagmin1	5.1 ± 0.3	7.1 ± 0.4*	1.3 (higher)
Inhibitory neurotransmitter receptors	GABA_A_R	4.9 ± 0.1	7.4 ± 0.3**	1.5 (higher)
	GABA_B_R	7.5 ± 0.4	2.2 ± 0.2**	0.3 (lower)
Excitatory neurotransmitter receptors (Glutamate receptors)	NMDAR	3.5 ± 0.2	6.1 ± 0.3**	1.7 (higher)
	AMPAR	2.9 ± 0.1	7.2 ± 0.3**	2.5 (higher)
	mGluR1	1.0 ± 0.3	4.2 ± 0.3**	4.2 (higher)
	mGluR5	2.1 ± 0.1	1.2 ± 0.4*	0.6 (lower)
	mGluR2	0.8 ± 0.2	1.9 ± 0.3*	2.4 (higher)
	mGluR3	0.3 ± 0.1	1.7 ± 0.4*	5.6 (higher)
	mGluR4	1.1 ± 0.1	2.9 ± 0.2**	2.6 (higher)
	mGluR7	2.1 ± 0.3	2.1 ± 0.2	1.0 (no change)
	mGluR8	2.9 ± 0.3	3.0 ± 0.1	1.0 (no change)

^a^ Ratio represents the relative expression level in IR-stimulated group compared to that in NGF-BDNF-stimulated group.

* p < 0.05,

** p < 0.01 vs NGF-BDNF-treated group

### Role of mGluR1 in the neuronal properties induced by IR

**Fig 4:**
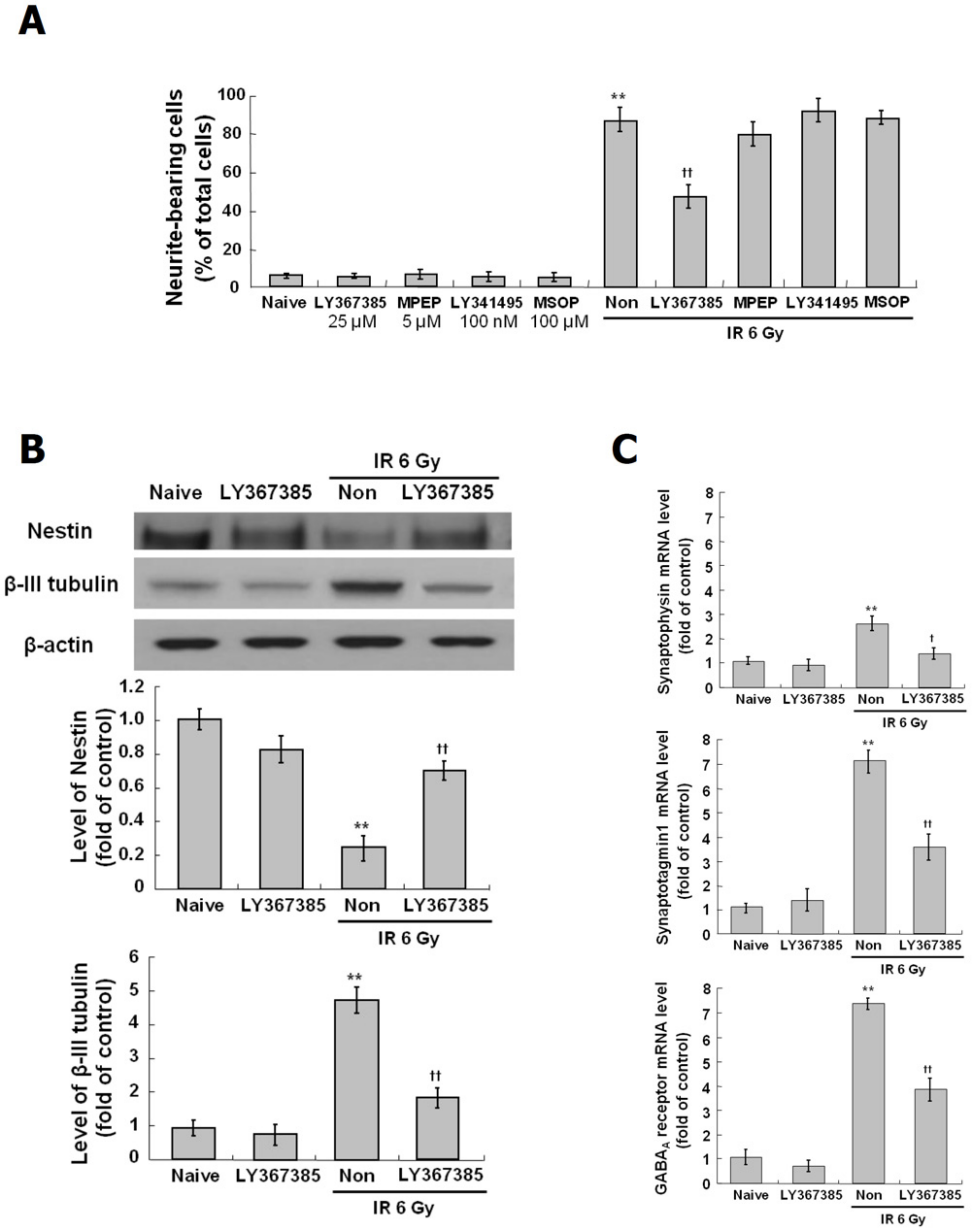
Suppression of IR-induced neuronal properties by mGluR1 antagonist, LY367385 in C17.2 cells. C17.2 cells treated with the antagonists of mGluR1 (LY367385) at 25 μM, mGluR5 (MPEP) at 5 μM, Group II mGluRs (mGluR2 and mGluR3; LY341495) at 100 nM and Group III mGluRs (mGluR4, mGluR7 and mGluR8; MSOP) at 100 μM for 2 hr, and then exposed to IR at 6 Gy and incubated at 37°C for 72 hr. To assess rate of neurite bearing cells, each 200 cells in three randomly taken images were analyzed by Image J software. IR-induced neurite outgrowth is not affected by the antagonists of mGluR5, Group II mGluRs nad Group III mGluRs but was blocked by mGluR1 antagonist, LY367385 (A). Expression of NSC marker, nestin, and neuronal marker, β-III tubulin was analyzed by Western blot (B, upper panel), and then quantified by Image J software (B, down panel). To assess effect of mGluR1 inhibition on IR-induced neuronal function-related genes, mRNA expression of synaptophysin, synaptotagmin1 and GABA_A_ receptors was analyzed by real-time PCR (C). *p < 0.05, **p < 0.01 vs naive group, †p < 0.05, ††p < 0.01 vs radiation only group.

To evaluate the role of mGluRs in the IR-induced neuronal differentiation, antagonists of mGluR1 (LY367385, 25 μM), mGluR5 (MPEP, 5 μM), Group II mGluRs (mGluR2 and mGluR3; LY341495, 100 nM) and Group III mGluRs (mGluR4, mGluR7 and mGluR8; MSOP, 100 μM) were applied to C17.2 cells prior to exposure to IR at 6 Gy and neurite outgrowth was examined. As shown in Fig 4A, the IR-induced neurite outgrowth was blocked only by the antagonist of mGluR1 (LY367385). We further investigated the effects of mGluR1 antagonist (LY367385) on IR-induced neuronal differentiation. The decreased expression of nestin in irradiated cells was partially restored by LY367385 (Fig 4B). The induction of β-III tubulin by IR was blocked by LY367385 (Fig 4B). The IR-induced expressions of synaptophysin, synaptotagmain 1 and GABA_A_ receptor were decreased by LY367385 (Fig 4C). These results suggest that mGluR1 plays a significant role in the IR-induced neuronal differentiation.

### Role of STAT3 and p53 in the neuronal differentiation and mGluR1 expression induced by IR

**Fig 5:**
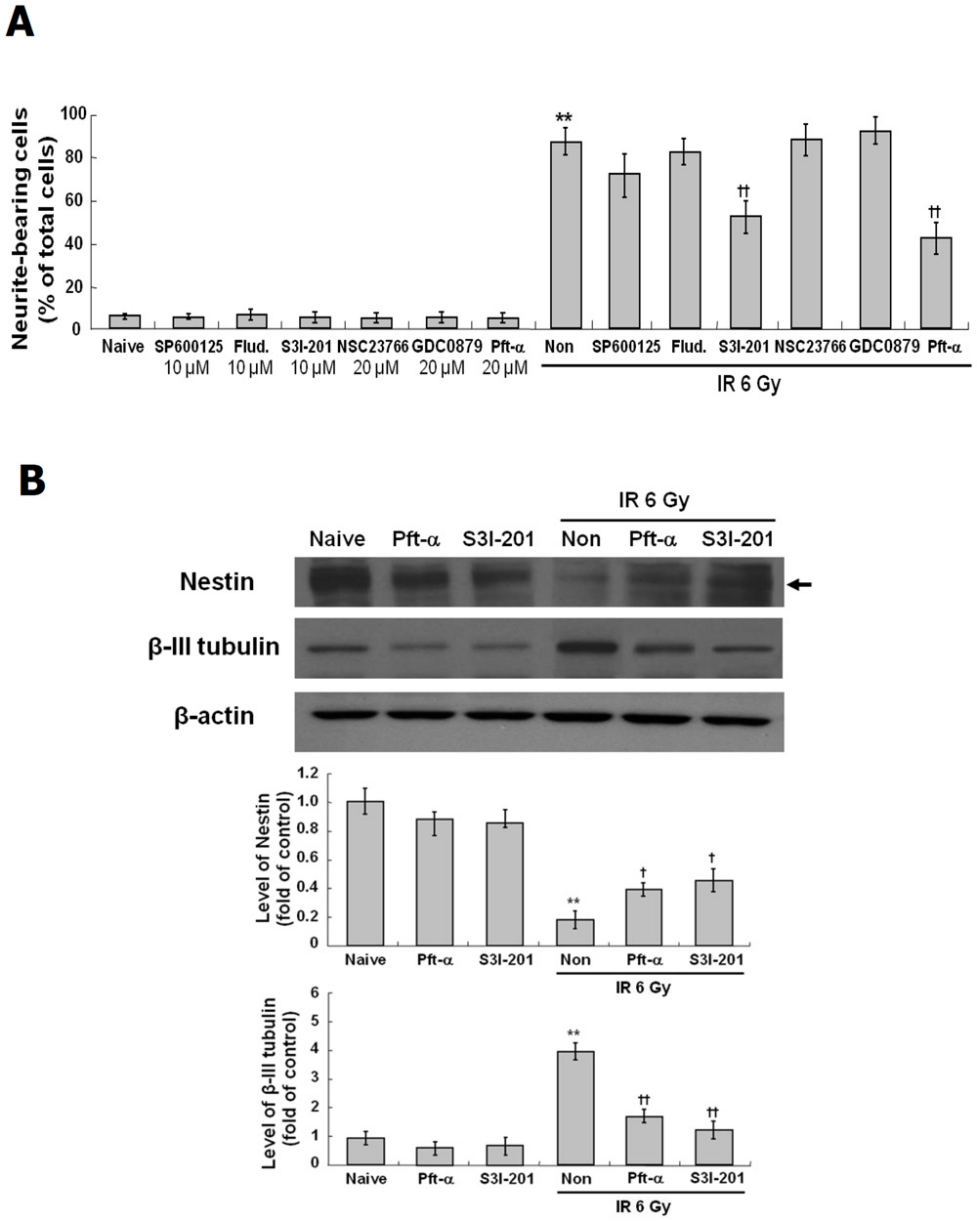
Effect of STAT3 and p53 inhibitors on IR-induced neuronal properties in C17.2 cells. C17.2 cells were treated with the inhibitors of JNK (SP600125) at10 μM, STAT1 (Fludarabine) at 10 μM, STAT3 (S3I-201) at 10 μM, Rac (NSC23766) at 20 μM, B-Raf (GDC-0879) at 20 μM and p53 (Pft-α) at 20 μM for 2 hr, and then exposed to IR at 6 Gy and incubated at 37°C for 72 hr. To assess rate of neurite bearing cells, each 200 cells in three randomly taken images were analyzed by Image J software. IR-induced neurite outgrowth is not affected by the inhibitors of JNK, STAT1, Rac and B-Raf but was blocked by the STAT3 inhibitor, S3I-201 and the p53 inhibitor, Pft-α (A). Expression of NSC marker, nestin, and neuronal marker, β-III tubulin was analyzed by Western blot (B, upper panel), and then quantified by Image J software (B, down panel). **p < 0.01 vs naive group, †p < 0.05, ††p < 0.01 vs radiation only group.

**Fig 6:**
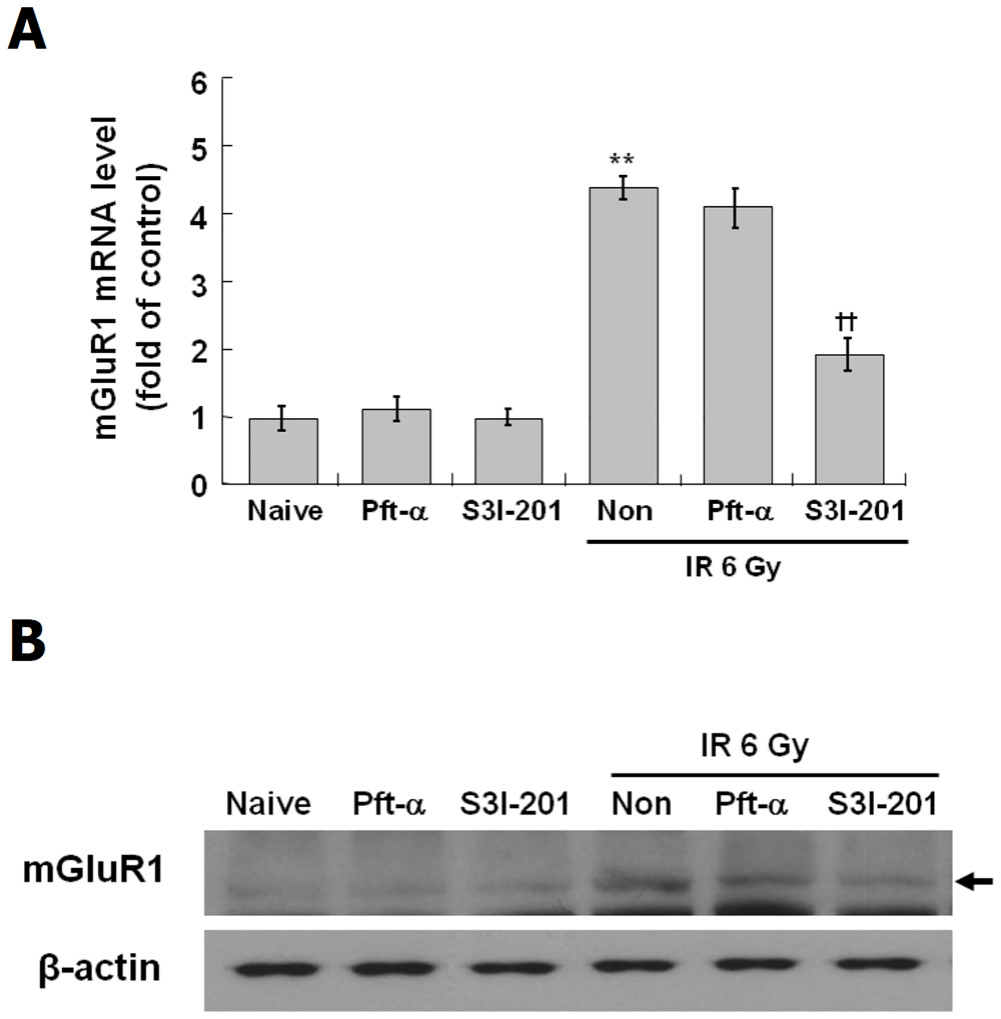
Suppression of IR-induced mGluR1 expression by STAT3 inhibitor in C17.2 cells. C17.2 cells were treated with STAT3 inhibitor, S3I-201 at 10 μM and p53 inhibitor, Pft-α at 20 μM for 2 hr, and then exposed to IR at 6 Gy and incubated at 37°C for 72 hr. Level of mGluR1 mRNA was analyzed by real-time PCR (A) and expression of mGluR1 was analyzed by Western blot (B). The results represent the mean ± SD from triplicate data. **p < 0.01 vs naive group, ††p < 0.01 vs radiation only group.

To identify other mediators of the IR-induced neuronal differentiation, the effects of inhibitors of various mediators known to be involved in neuronal differentiation and IR responses was examined. C17.2 cells were treated with inhibitors of JNK (SP600125, 10 μM), STAT1 (Fludarabine, 10 μM), STAT3 (S3I-201, 10 μM), Rac (NSC23766, 20 μM), B-Raf (GDC-0879, 20 μM) and p53 (Pft-α, 20 μM) prior to exposure to IR at 6 Gy. As shown in Fig 5A, the induction of neurite outgrowth by IR was partially blocked by S3I-201 and Pft-α but was not changed by inhibitors of JNK, STAT1, Rac or B-Raf. As shown in Fig 5B, the decreased expression of nestin was partially restored and the elevated expression of β-III tubulin was blocked by STAT3 and p53 inhibitors. Next, the effects of STAT3 or p53 on the IR-induced mGluR1 expression were evaluated in C17.2 cells treated with S3I-201 or Pft-α. As shown in Fig 6. IR-induced expressions of mGluR1 mRNA and protein were markedly decreased by STAT3 inhibitor but were not affected by p53 inhibitor, suggesting that the expression of mGluR1 is regulated by STAT3 signaling but not by p53 signaling. These results suggest that the IR-induced neuronal differentiation is mediated by STAT3-mGluR1 signal pathways and p53 signaling pathways.

### Role of PI3K in the neuronal marker expressions and STAT3-mGluR1 signaling induced by IR

**Fig 7:**
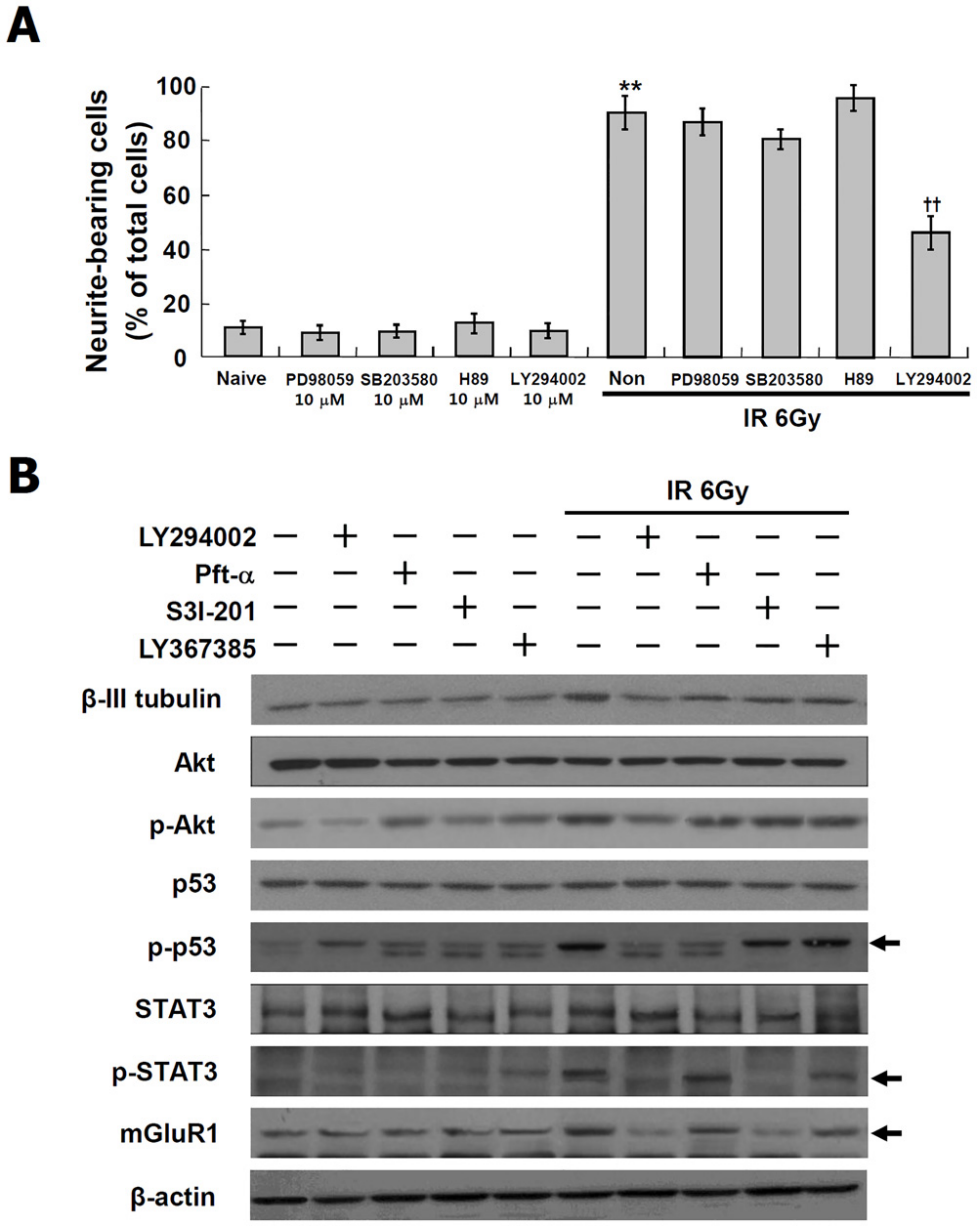
Suppression of IR-induced neuronal differentiation signaling by PI3K inhibitor in C17.2 cells. C17.2 cells treated with the inhibitors of MEK (PD98059), p38 (SB203580), PKA (H-89) and PI3K (LY294002) at 10 μM for 2 hr, and then exposed to IR at 6 Gy and incubated at 37°C for 72 hr. To assess rate of neurite bearing cells, each 200 cells in three randomly taken images were analyzed by Image J software. IR-induced neurite outgrowth is not affected by inhibitors of MEK, p38 and PKA but was blocked by PI3K inhibitor, LY294002 (A). Level of β-III tubulin and mGluR1, and activation of AKT, p53 and STAT3 were analyzed by Western blot (B). The results represent the mean ± SD from triplicate data. **p < 0.01 vs naive group, ††p < 0.01 vs radiation only group.

To confirm the upstream signals on STAT3-mGluR1, the effects of protein kinases were examined in irradiated C17.2 cells. The cells were treated with the inhibitors of MEK (PD98059), p38 (SB203580), PKA (H-89) and PI3K (LY294002) at 10 μM prior to the exposure to IR at 6 Gy. As shown in Fig 7A, the induction of neurite outgrowth by IR was significantly blocked by PI3K inhibitor but was not affected by inhibitors of MEK, PKA and p38. Next, the effects of PI3K inhibition on neuronal marker expression and PI3K-AKT, STAT-3-mGluR1 and p53 signaling in the irradiated C17.2 cells were investigated. As shown in Fig 7B, the IR-induced expression of β-III tubulin and mGluRl, and the IR-induced phophorylation of AKT, p53 and STAT3 were blocked by LY294002. These results suggest that PI3K regulates IR-induced neuronal differentiation in the upstream of STAT3-mGluR1 pathway and p53 pathway.

### Immunocytochemistry analysis on IR-induced neuronal differentiation through STAT3 and mGluR1 signaling

**Fig 8:**
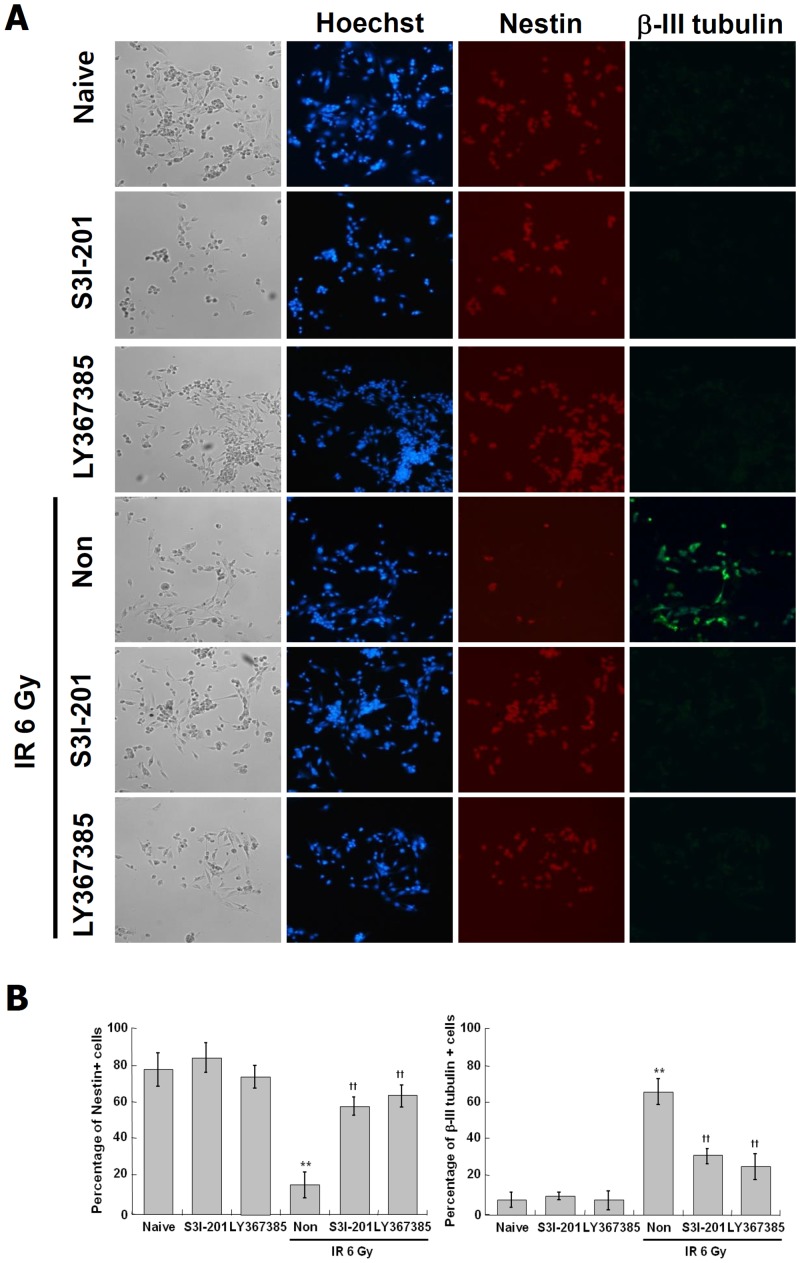
Effect of STAT3 inhibitor and mGluR1 antagonist on the IR-induced expression of neuronal markers in C17.2 cells. C17.2 cells were treated with S3I-201 at 10 M and LY367385 25 M at for 2 hr, and then exposed to IR at 6 Gy and incubated at 37°C for 72 hr. NSC marker, nestin positive (red) and neuronal marker, β-III tubulin positive (green) cells were analyzed by immunocytochemistry (×100 magnification) (A). Nuclei were labeled with Hoechst (blue). The rate of antibody positive cells was quantified by Image J software (B). **p < 0.01 vs naive group, ††p < 0.01 vs radiation only group.

To confirm the induction of neuronal differentiation by IR and the role of STAT3 and mGluR1 in this process, the expression of β-III tubulin and nestin were observed directly in the individual cell level using immunocytochemistry (ICC) analysis. IR increased markedly β-III tubulin positive cells and decreased dramatically the nestin positive cells, and S3I-201 or Pft-α almost completely suppressed the effects of IR (Fig 8). These results further confirmed that the IR-induced neuronal differentiation is mediated by STAT3 and mGluR1 in C17.2 cells.

### STAT3 and mGluR1 signaling in IR-induced neuronal differentiation in mouse primary neural stem cells and in

**Fig 9:**
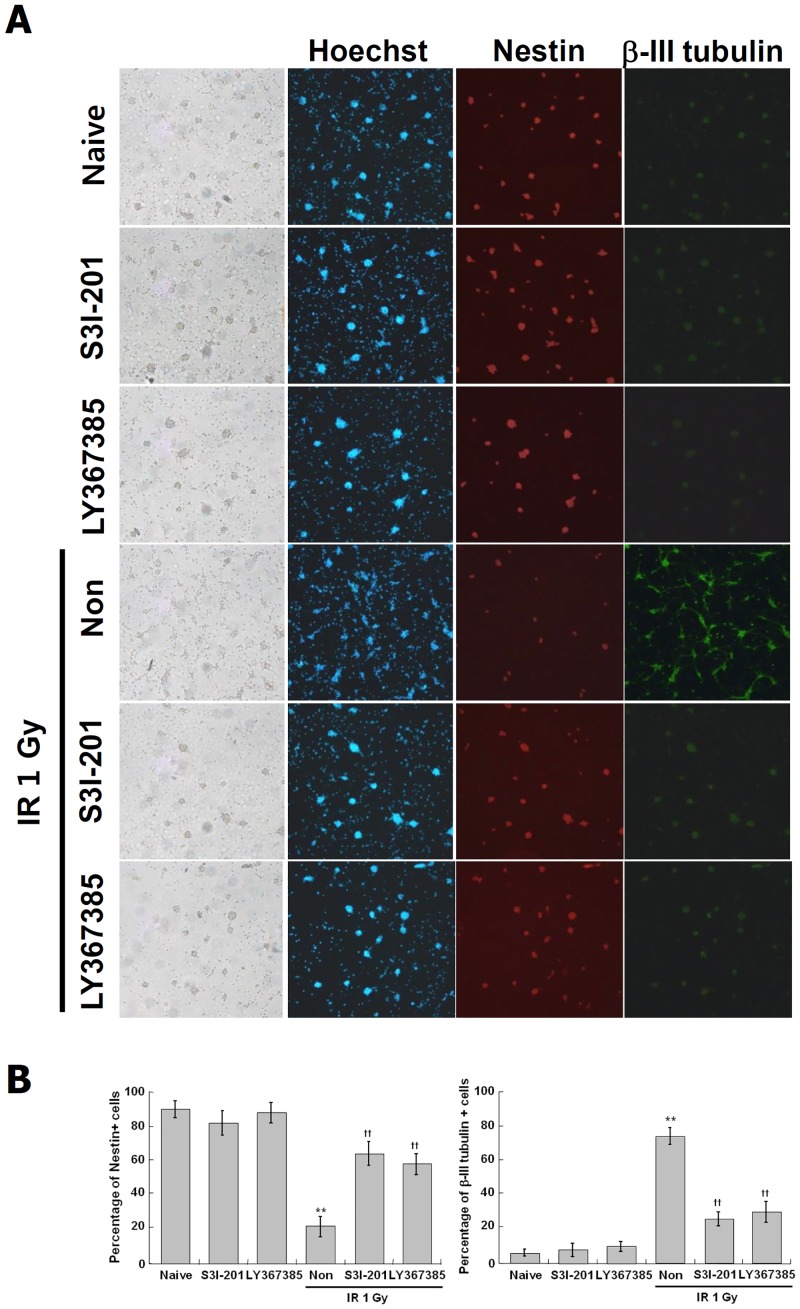
Effect of STAT3 inhibitor and mGluR1 antagonist on the IR-induced neuronal markers in mouse primary neural stem cells. Isolated NSCs were treated with S3I-201 at 5 M and LY367385 10 M at for 2 hr, and then exposed to IR at 1 Gy and incubated at 37°C for 72 hr. NSC marker, nestin positive (red) and neuronal marker, β-III tubulin positive (green) cells were analyzed by immunocytochemistry (×100 magnification) (A). Nuclei were labeled with Hoechst (blue). The rate of antibody positive cells was quantified by Image J software (B). **p < 0.01 vs naive group, ††p < 0.01 vs radiation only group.

Mouse primary NSCs isolated from hippocampus of newborn mice were used to verify the IR-induced neuronal differentiation and the role of STAT3 and mGluR1 in this process. Primary NSCs were exposed to IR at 0.5~8 Gy, and incubated for 72 hr. The growth of cells was decreased by IR in a dose-dependent manner (data not shown). Because the viability of cells was sharply decreased at 2 Gy or higher doses, radiation dose at 1 Gy was used in subsequent experiments. To confirm the role of STAT3 and mGluR1 in the IR-induced neuronal differentiation of primary NSC, the cells were treated with S3I-201 at 5 μM and LY367385 at 10 μM prior to exposure to IR at 1 Gy, incubated for 72 hr, and analyzed for NSC and neuronal markers by ICC. In accordance with the results of C17.2 cells, βIII tubulin-positive cells were increased and nestin-positive cells were decreased in the irradiated primary NSCs (Fig 9). These effects were blocked by STAT3 inhibitors and mGluR1 antagonist (Fig 9). These results suggest that the IR can induce neuronal differentiation also in primary NSCs and mGluR1 and STAT3 play a role as mediators of this process.

## Discussion

Radiotherapy for treatment of primary and metastatic brain tumors is often accompanied by acute adverse effects, such as sickness, emesis, headache, vertigo and seizures, and late adverse effects such as cognitive deficits and memory loss [3]. Brain exposure to radiation in animal models causes chronic inflammation by abnormal increases of cytokines and activated microglia, and subsequent cognitive loss resulting from injury of the hippocampus which is a key area for learning and memory [11, 33]. In addition, radiation is known to directly affect NSCs which are a decisive factor of neurogenesis and neuronal repair by inducing ROS generation, cell death and an inhibition of neuronal differentiation [34, 35]. However, some studies have reported contrary results that irradiation at 8 Gy to neuronal precursor cells isolated from newborn mouse caused no significant changes in neuronal differentiation [61] and NSCs prepared from mouse embryonic stem cells retained abilities to proliferate and differentiate when exposed to 1 Gy of X-ray [36]. Although a number of studies have focused on the damage to neuronal cells and/or tissues caused by radiation *in vitro* and *in vivo*, several studies reported the influence of IR on neuronal differentiation by showing that an exposure to X-ray induced the neurite outgrowth in PC-12 cells [62] and the astrocyte-specific differentiation in NSCs and mouse brain [37, 38, 63]. However, the effects of IR on neuronal differentiation are largely unknown.

In our previous study, we observed that undifferentiated Neuro-2a neuroblastoma cells exposed to a non-lethal dose of radiation showed neuron-like morphological and molecular changes, such as neurite formation and neuronal marker expression [58]. However, Neuro-2a cells are derived from neuroblastoma and their responses to IR can be different from those of NSCs in the brain. Thus, this study aimed to examine whether IR can induce neuronal differentiation in C17.2 cells, an immortalized and multipotent neural stem-like cell line derived from mouse cerebellum, and in mouse primary neural stem cells, and to identify the underlying mechanisms. In addition, the neuronal properties of irradiated C17.2 cells were compared to those of cells differentiated by neurotrophin treatment to examine whether both differentiation stimuli induces similar or different patterns of differentiation to a large extent. The responses of C17.2 cells to IR were similar to the results of our previous study with Neuro-2a cell [58]. Neurite outgrowth and mRNA of GAP43 and Rab13 were increased by IR, and similar results were also observed by NGF/BDNF-treatment (Fig 1). An increase of β-III tubulin and a decrease of nestin in both irradiated cells and NGF/BDNF-treated cells indicated the neuronal differentiation from neural stem cells (Fig 2). Increased neuronal marker expression in irradiated C17.2 cells was also observed by ICC (Fig 8). Although, some studies have reported that astrocytic differentiation was increased by X-ray in NSCs [37, 38], the astrocyte marker, GFAP expression was not changed in both groups (Fig 2). The discrepancy seems to be attributed to various factors that can affect the differentiation of NSC including the origin of NSCs, radiation dose and dose rate, and culture condition. However, our results clearly showed that, at least in our experimental conditions, IR can induce neuronal properties without pharmacological stimulation in neural stem-like cells.

The major function of neuron is to construct a synapse which is a passage for transmission of electrical or chemical signal to another neuron [64]. The differentiated neuron expresses various synapse-related proteins, such as synaptophysin, synaptotagmin1, GABA receptors and glutamate receptors, involving the formation of synaptic vesicle and the responses to calcium ion or neurotransmitters [65–70]. Therefore, the neuronal function of C17.2 cells differentiated by IR was confirmed by examining the expressions of neuronal function-related synaptophysin, synaptotagmin1, GABA receptors and glutamate receptors. As shown in Fig 3, IR increased the expression of synaptophysin, synaptotagmin1, GABA receptors and glutamate receptors in C17.2 cells. However, the expression pattern of the glutamate receptors in irradiated cells was significantly increased in irradiated cells than NGF/BDNF-treated cells (Fig 3C and 3D, and Table 3). These results show that the IR-induced neuronal differentiation of C17.2 cells may lead to altered neuronal function with an elevated excitatory synaptic response. Other study has reported that acute irradiation increased the expression of GABA_A_ receptors and decreased the expression of glutamate receptor in hippocampal brain slices [71]. Although their results conflict with our result, it is likely to be due to the fact that their results focused on acute response that occurred within 30 min after irradiation at high dose (10 Gy) in both differentiated neurons and NSCs of DG. Also, some studies have reported that the seizures, one of adverse effects of radiotherapy, is caused by change of electrical impulse through the loss of GABA receptors or the abnormal increase of calcium ion channels [72, 73]. From the results of this study and previous reports, it is possible that the abnormal changes of synaptic signaling may be associated with IR-induced brain injuries. However, the comparison of IR-induced and neurotrophin-induced differentiation in a cell-based model cannot be decisive since these differences can be dependent on the specific experimental conditions including IR or neurotrophin doses, and time points.

The various glutamate receptors mediate glutamate signaling for several excitatory synaptic responses [49, 67]. However, recent studies have reported that the mGluRs are involved in survival and proliferation of NPC, regulation of hippocampal development and differentiation of NSC [52–57]. Thus, the association of mGluRs with the IR-induced neuronal properties was investigated using their antagonists in C17.2 cells. The antagonist of mGluR1 signaling decreased IR-induced neurite outgrowth, neuronal marker (β-III tubulin) and neuronal function-related synatpophysin, synaptotagmin1 and GABA_A_ receptor, while the antagonists of other mGluRs were not effective (Figs 4 and 8). These results show that the IR-induced neuronal differentiation is regulated by mGluR1 in C17.2 cells.

To identify upstream signals of mGluR1, we examined the involvement of various signaling molecules associated with neuronal differentiation and IR response-related signals [74–82]. As shown in Figs 5 and 8, IR-induced neurite outgrowth and neuronal marker (β-III tubulin) expression were decreased by the inhibition of STAT3 or p53. Also, the mRNA and protein expression of mGluR1 were suppressed by STAT3 inhibition, but not by p53 inhibition (Fig 6). Although p53 played a role in IR-induced neuronal properties in C17.2 cells (Fig 5), mGluR1-related signaling in IR-induced differentiation was independent of p53 and dependent on STAT3. These results show that the IR-induced neuronal differentiation may be mediated by STAT3-mGluR1 pathway in C17.2 cells. A recent study reported that the activation of STAT1 and STAT3 leads to expression of GAP-43, β-III tubulin and synaptophysin [83], which supports the results of this study. However, another group has reported that differentiation of motor neuron was inhibited by STAT3 in human NSC [84]. These conflicting results may be due to the fact that STAT3 play a role as a transcription factor involved in a wide variety of cellular signaling mechanisms

Then the upstream signals of STAT3-mGluR1 were examined. As demonstrated in our previously study, the induction of neuronal properties was mediated by PI3K-p53 signaling in Neuro-2a cells [58]. It was also reported that an interaction between PI3K and mGluR1 is involved in prevention of neuronal apoptosis [85]. In addition, neurite outgrowth and neuronal differentiation by retinoic acid (RA) or neurotophins are reported to be mediated by MEK, PKA and p38 as well as PI3K [74, 75, 86–90]. Therefore, the effects of these molecules on IR-induced neuronal properties and STAT3-mGluR1 signaling were examined to identify the upstream signals in irradiated C17.2 cells. Inhibition of PI3K signaling decreased neurite outgrowth, the expression of β-III tubulin and mGluR1, and the phosphorylation of AKT, p53 and STAT3 induced by IR (Fig 7). These results suggest that PI3K acts in the upstream of STAT3-mGluR1 in the IR-induced neuronal differentiation.

Finally, the IR-induced neuronal differentiation observed in C17.2 cells was verified in *ex vivo* experiment using mouse primary NSCs to ensure the *in vivo* relevance. IR induced the elevation of β-III tubulin positive cells and the decline of nestin positive cells and these changes were blocked by inhibition of STAT3 or mGluR1 in primary NSCs (Fig 9), showing similar results as in C17.2 cells. These results suggest that the phenomenon of the IR-induced neuronal differentiation may also occur *in vivo*. However, *in vivo* examination of neuronal differentiation and its related signals should be conducted in the future study to confirm the *in vivo* relevance of the IR-induced neuronal differentiation.

**Fig 10:**
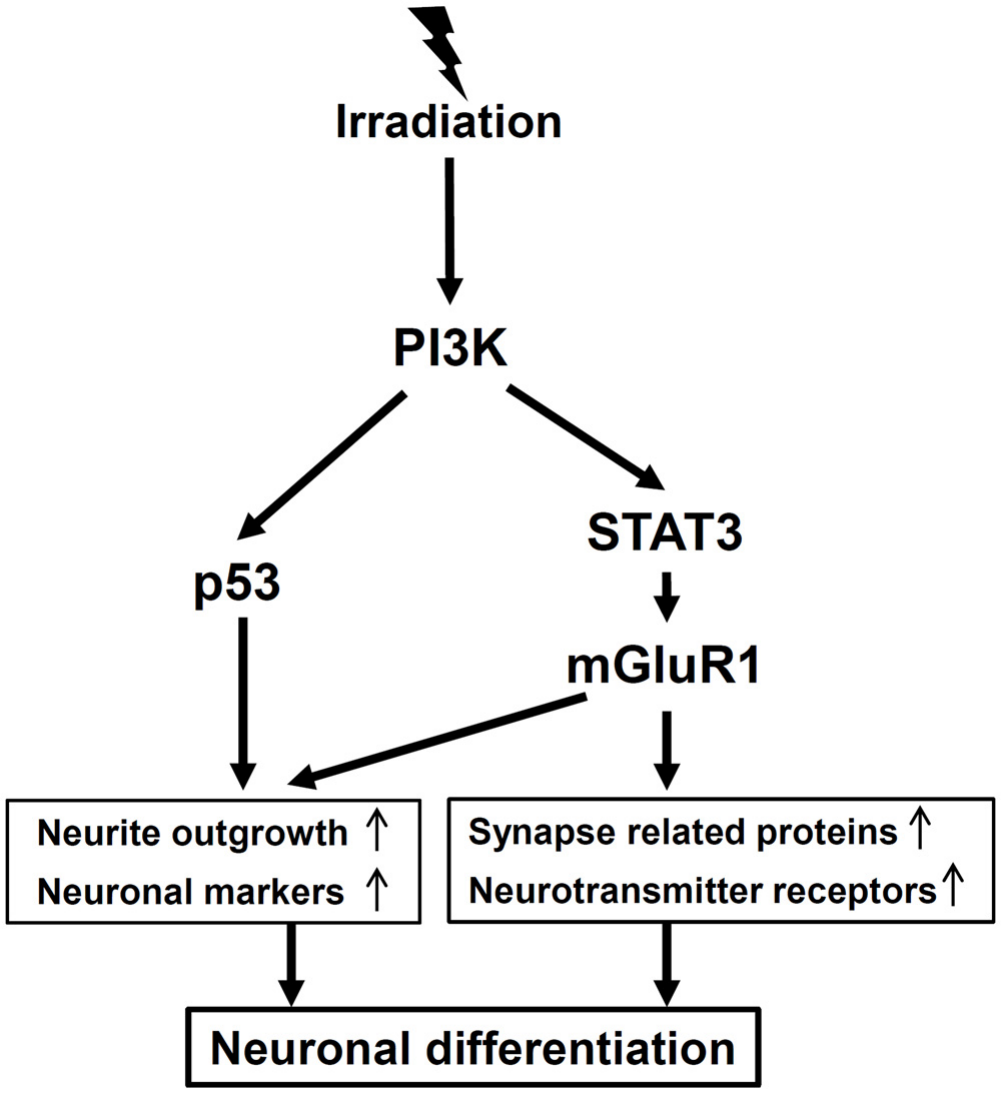
Schematic representation of signaling pathway in IR-induced neuronal differentiation. Ionizing radiation promotes the neuronal differentiation of C17.2 cells via two independent signaling pathways, PI3K-p53 and PI3K-STAT3-mGluR1. p53 and STAT3 may play as the downstream target of PI3K signaling in IR-induced neuronal differentiation of C17.2 cells. The activation of p53 via PI3K may be involved in the IR-induced neurite outgrowth and neuronal marker expressions. mGluR1 signaling of PI3K-STAT3 downstream may also be involved in the IR-induced neuronal function-related protein expressions as well as neurite outgrowth and neuronal marker expressions.

To summarize the results of this study, the neuronal properties, including neurite outgrowth, neuronal marker, β-III tubulin, and neuronal function-related proteins, synaptophysin, synaptotagmin1, GABA receptors and glutamate receptors, were induced by IR. The induction of neuronal properties by IR was mediated by two pathways, i.e., PI3K-STAT3-mGluR1 and PI3K-p53 signaling pathways (Fig 10). PI3K regulated the activation of both pathways, but STAT3-mGluR1 and p53 in the downstream did not affect each other (Figs 7 and 10). IR-induced neuronal differentiation exhibited similar properties overall as neurotrophin-induced differentiation. However, slight different expression profiles of neuronal function-related genes were observed between IR- and neurotrophin-induced differentiations. IR-induced differentiation showed higher expression of excitatory neurotransmitter receptors, suggesting that it may lead to altered neuronal functions, especially in excitatory synaptic responses. To overcome adverse effects of radiotherapy on brain function, most of the studies have tried to reduce NSC loss caused by direct or indirect cellular damage induced by IR responses. However, recent studies reported by others and this study suggest that the control of abnormal differentiation as well as NSC loss may be required to completely overcome the advers25e effects by brain radiotherapy. In this point of view, the results of this study may provide an important clue that IR-induced neuronal differentiation can affect the neurogenesis and cognitive function. However, further *in vivo* studies using cranial irradiation should be performed to verify whether neuronal differentiation of NSC is actually induced by IR mediated by PI3K, p53, STAT3 and mGluR1 in the brain, and whether the neuronal differentiation exerts the adverse or beneficial effects in the brain function. Investigations of IR-induced neuronal differentiation may contribute to the understanding of the acute and chronic effects of IR on brain functions and to the development of therapeutic strategies to reduce degenerative damages by IR during brain radiotherapy.

## Supporting Information

S1 Fig Cell viability of irradiated C17.2 cells. C17.2 cells were γ-irradiated at 0, 2, 4, 8, 16 Gy. After 72 hr, the viability of C17.2 cells was analyzed colorimetrically with WST-8 reagent. The results represent the mean ± SD from triplicate data. *p < 0.05, **p < 0.01 vs naïve group.
(TIF)

S2 Fig Cell viability of irradiated primary NSCs. Mouse primary neural stem cells were γ-irradiated at 0, 0.5, 1, 2, 4, 8 Gy. Cells were incubated for 72 hr and the cell viability was analyzed colorimetrically with WST-8 reagent. The results represent the mean ± SD from triplicate data. *p < 0.05, **p < 0.01 vs naïve group.
(TIF)

S3 Fig Effects off IR on the neurite outgrowth of C17.2 cells treated with neurotrophins. C17.2 cells were γ-irradiated at 0 or 8 Gy, and then incubated for 72 hr in the absence or presence of NGF and BDNF. The morphological change for neurite outgrowth was observed in microscopic images (×200 magnification) (A). To assess the rate of neurite-bearing cells, each 200 cells in three randomly taken images were analyzed by Image J software (B). The results represent the mean ± SD from triplicate data. *p < 0.05, **p < 0.01 vs 0Gy group.
(TIF)
