# Supplementary figures and images for: Ionizing Radiation Induces Altered Neuronal Differentiation by mGluR1 through PI3K-STAT3 Signaling in C17.2 Mouse Neural Stem-Like Cells
Source: PLoS One. 2016 Feb 1;11(2):e0147538. doi: 10.1371/journal.pone.0147538 (PMC4734671; doi:10.1371/journal.pone.0147538)

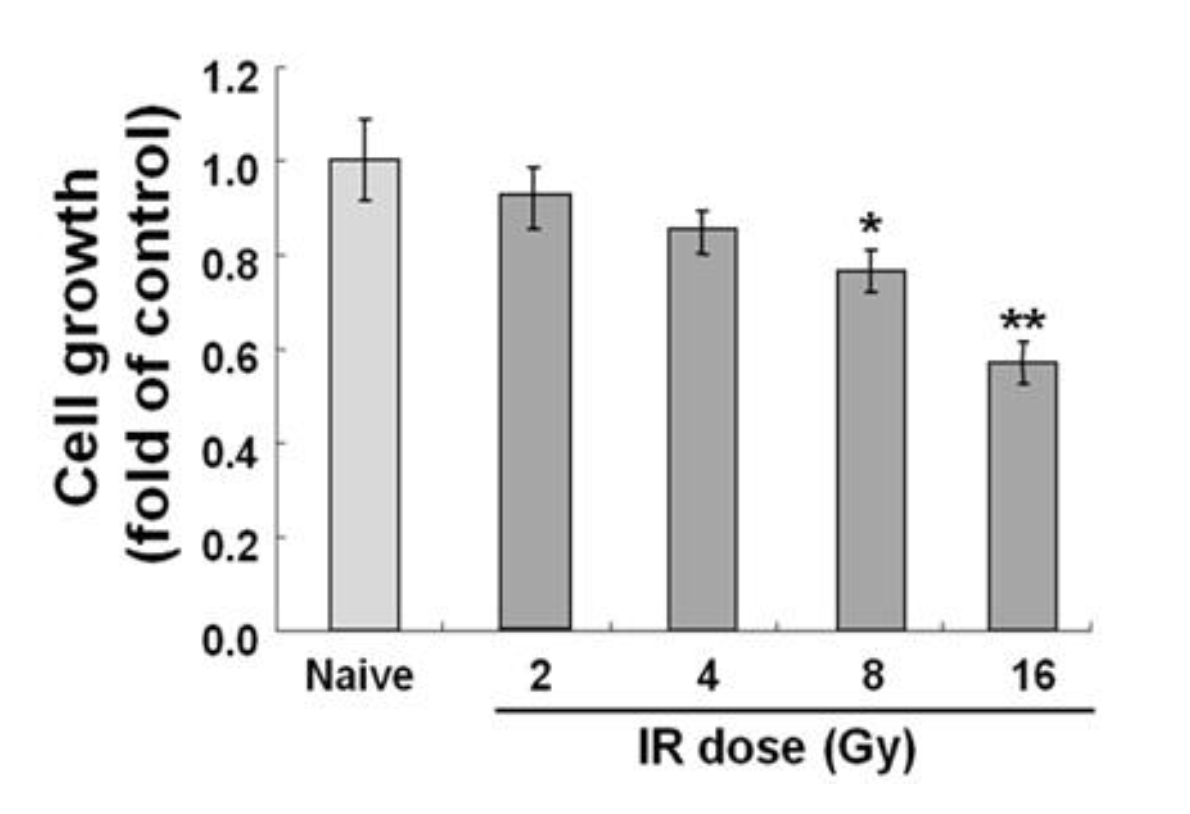

Supplement: S1 Fig — C17.2 cells were γ-irradiated at 0, 2, 4, 8, 16 Gy. After 72 hr, the viability of C17.2 cells was analyzed colorimetrically with WST-8 reagent. The results represent the mean ± SD from triplicate data. *p < 0.05, **p < 0.01 vs naïve group. (TIF) [file pone.0147538.s001.tif]

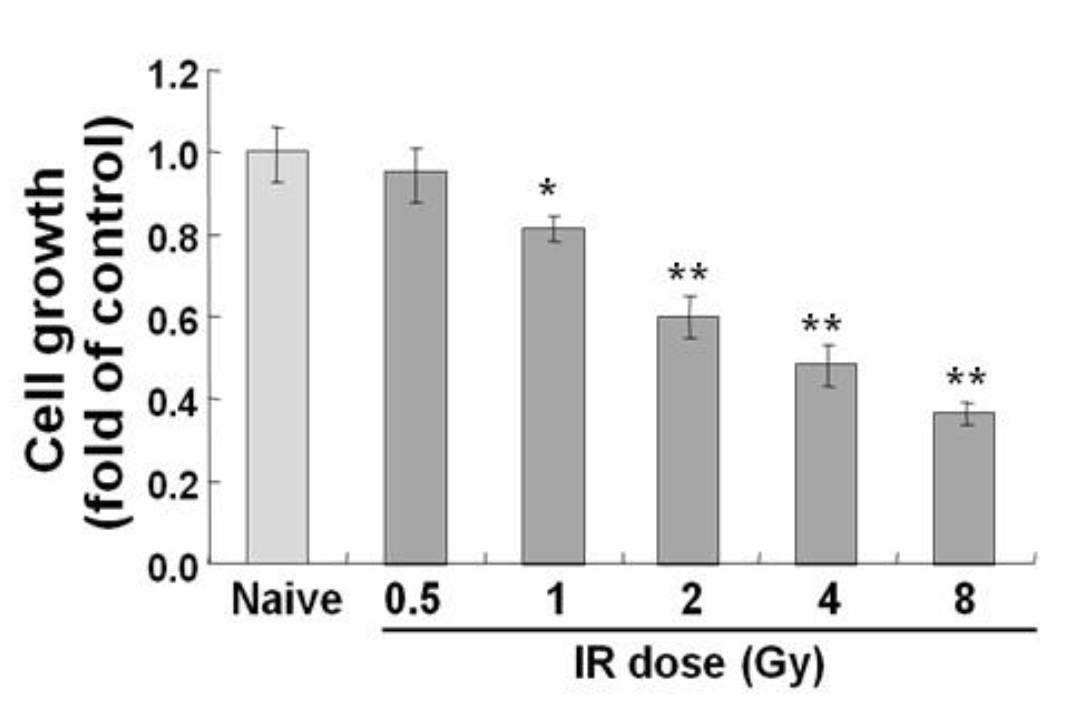

Supplement: S2 Fig — Mouse primary neural stem cells were γ-irradiated at 0, 0.5, 1, 2, 4, 8 Gy. Cells were incubated for 72 hr and the cell viability was analyzed colorimetrically with WST-8 reagent. The results represent the mean ± SD from triplicate data. *p < 0.05, **p < 0.01 vs naïve group. (TIF) [file pone.0147538.s002.tif]

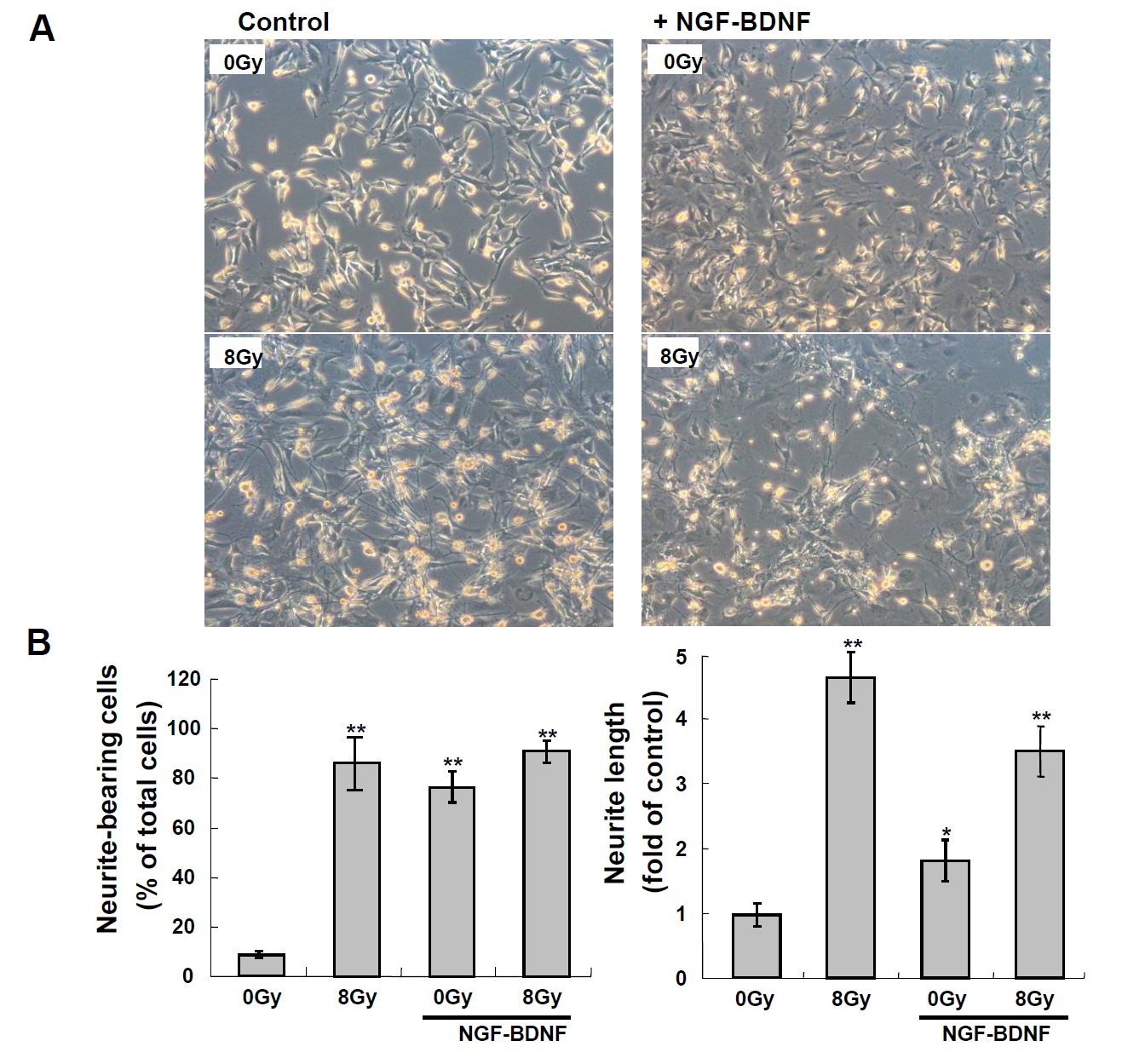

Supplement: S3 Fig — C17.2 cells were γ-irradiated at 0 or 8 Gy, and then incubated for 72 hr in the absence or presence of NGF and BDNF. The morphological change for neurite outgrowth was observed in microscopic images (×200 magnification) (A). To assess the rate of neurite-bearing cells, each 200 cells in three randomly taken images were analyzed by Image J software (B). The results represent the mean ± SD from triplicate data. *p < 0.05, **p < 0.01 vs 0Gy group. (TIF) [file pone.0147538.s003.tif]
